# Rapid Detection and Quantification of Triacylglycerol by HPLC–ELSD in *Chlamydomonas reinhardtii* and *Chlorella* Strains

**DOI:** 10.1007/s11745-013-3828-9

**Published:** 2013-08-23

**Authors:** Naoko Kobayashi, Eric A. Noel, Austin Barnes, Julian Rosenberg, Concetta DiRusso, Paul Black, George A. Oyler

**Affiliations:** 1 Department of Biochemistry, University of Nebraska-Lincoln, 1901 Vine Street, Lincoln, NE 68588 USA; 2 School of Biological Sciences, University of Nebraska-Lincoln, 1104 T Street, Lincoln, NE 68588 USA; 3 Department of Chemical and Biomolecular Engineering, Johns Hopkins University, 3400 North Charles Street, Baltimore, MD 21218 USA

**Keywords:** TAG quantification, HPLC–ELSD, GC–MS, TLC, *Chlamydomonas reinhardtii*, *Chlorella*, Lipid, FAME, Lipid class

## Abstract

Triacylglycerol (TAG) analysis and quantification are commonly performed by first obtaining a purified TAG fraction from a total neutral lipid extract using thin-layer chromatography (TLC), and then analyzing the fatty acid composition of the purified TAG fraction by gas chromatography (GC). This process is time-consuming, labor intensive and is not suitable for analysis of small sample sizes or large numbers. A rapid and efficient method for monitoring oil accumulation in algae using high performance liquid chromatography for separation of all lipid classes combined with detection by evaporative light scattering (HPLC–ELSD) was developed and compared to the conventional TLC/GC method. TAG accumulation in two *Chlamydomonas reinhardtii* (21 gr and CC503) and three *Chlorella* strains (UTEX 1230, CS01 and UTEX 2229) grown under conditions of nitrogen depletion was measured. The TAG levels were found to be 3–6 % DW (*Chlamydomonas* strains) and 7–12 % DW (*Chlorella* strains) respectively by both HPLC–ELSD and TLC/GC methods. HPLC–ELSD resolved the major lipid classes such as carotenoids, TAG, diacylglycerol (DAG), free fatty acids, phospholipids, and galactolipids in a 15-min run. Quantitation of TAG content was based on comparison to calibration curves of trihexadecanoin (16:0 TAG) and trioctadecadienoin (18:2 TAG) and showed linearity from 0.2 to 10 μg. Algal TAG levels >0.5 μg/g DW were detectable by this method. Furthermore TAG content in *Chlorella kessleri* UTEX 2229 could be detected. TAG as well as DAG and TAG content were estimated at 1.6 % DW by HPLC–ELSD, while it was undetectable by TLC/GC method.

## Introduction

Microalgae serve as a potential feedstock for biofuel and biodiesel applications due to the ability of some algal strains to accumulate high levels of triacylglycerols (TAG) under various stress conditions [1]. TAG are neutral lipids consisting of a glycerol backbone esterified to three fatty acids. The fatty acid composition of algal TAG is similar to that observed in higher plants, with 16- and 18-carbon fatty acids being predominant [1, 2]. Most conventional fuels including petroleum and diesel contain aliphatic hydrocarbons that are chemically similar to the fatty acid components of TAG [2]. Algal TAG accumulation has been studied under conditions of nutrient limitation and abiotic stress to monitor changes in compound production [1]. These studies include gene expression patterns and their relation to oil accumulation during nitrogen deprivation in *C. reinhardtii* and *Coccomyxa* sp. [3], lipid profiling during photoautotrophic and heterotrophic growth in *Chlorella zofingiensis* [4] and characterization of changes in major lipid droplet proteins in response to nitrogen deprivation in *C. reinhardtii* [5].

Thin layer chromatography (TLC) and gas chromatography (GC) are the most commonly used analytical methods for lipid characterization and quantification. The combination of methods involves purifying specific lipid classes such as TAG first by TLC, scraping individual bands from the TLC plate, extracting these bands, hydrolyzing the fatty acids to form fatty acid methyl esters (FAME), and finally analyzing the FAME by gas chromatography. The separation of lipid classes by silica TLC [3–5] or silica column [6] requires use of different solvent systems for purification of each lipid class. TAG are well separated from other major lipid classes such as phospholipids and glycolipids in a non-polar solvent such as chloroform, hexane or heptane, however phospholipids and glycolipids cannot be resolved in highly non-polar solvent systems and require more polar solvent systems. After TAG isolation by TLC, intact TAG must be broken down to release the fatty acid components which are converted to FAME by a trans-esterification reaction in preparation for GC analysis. Accurate quantitation by TLC/GC involves a number of purification steps and a trans-esterification reaction which leads to an indirect measurement of the total lipid content. Although the fatty acid compositions by traditional TLC and GC methods are useful for qualitative and quantitative lipid analyses, sample preparation is time-consuming, labor-intensive and not suitable for profiling total lipid compositions in a high-throughput manner.

Here we describe a method using high performance liquid chromatography–evaporative light scattering detector (HPLC–ELSD) that is suitable for comparing major lipid classes in a single injection and can be used to accurately measure TAG content. HPLC–ELSD involves separation of lipids by HPLC, and detection by conversion to small particles in a nitrogen gas stream and collision with light. Separation and quantification of different lipid classes by HPLC–ELSD have been reported in various species. For example, nine TAG from Walnut (*Juglans regia* L.) were separated and identified by HPLC–ELSD including trilinolein, dilinoleoyl-oleoyl-glycerol and dilinolenoyl-glycerol as the major TAG at 38, 19 and 18 %, respectively [7]. In addition, ten complex lipid classes were separated with a binary gradient system by HPLC–ELSD and glyceroglycolipids including monogalactosyldiacylglycerol (MGDG), digalactosyldiacylglycerol (DGDG) and sulfoquinovosyldiacylglycerol (SQDG) were quantified simultaneously in different plants [8] and 17 neutral and polar lipid classes were separated by HPLC–ELSD using a monolithic silica phase and quantified in four different zooplankton [9]. A 15 min HPLC gradient with hexane based solvent and isopropanol based solvent is used to resolve carotenoids, TAG, DAG and free fatty acids, and the major membrane components: phosphophatidylcholine (PtdCho), MGDG and DGDG. TAG content is estimated based on comparison to calibration curves of trihexadecanoin (16:0 TAG) and trioctadecadienoin (18:2 TAG). *C. reinhardtii* has been established as the model green microalgae with TAG contents ranging widely from 2 to 20 % of DW in different strains by TLC/GC method [10]. Although HPLC is mostly tandem with UV–VIS or MS, ELSD is more accurate lipid quantification than UV–VIS and more inexpensive and easier than MS. The advantage of using ELSD is to make it possible to quantify not only TAG but other neutral lipids for biofuel or biodiesel at the same run, while it requires several steps and different GC runs by TLC/GC method.

The green microalgae of the genus *Chlorella* are among the potential candidates of biofuel or biodiesel production due to inherent characteristics of large biomass production and oil accumulation with minimal nutrient requirements [11]. *Chlorella* cultivation can be readily performed in industrial processes [12, 13]. Reported oil accumulation of *Chlorella* spans broadly from 3 to 58 % DW, illustrating a wide range of sensitivity by various methods of extraction, analysis and quantification [14–17]. TAG analysis has been reported commonly in *Chlorella* by gravimetric measurement, or by GC with TLC or column chromatography in *C. vulgaris*, *C. zofingiensis* and *C. sorokiniana* [18–20]. Further means of TAG detection have included Raman spectroscopy in *C. sorokiniana* [21], thermogravimetric analysis in *Chlorella* sp. [22] and HPLC–ELSD in *C. minutissima* [23]. Using HPLC–ELSD, contents of TAG in a variety of algae under different conditions were screened to select a candidate species best suitable for yielding high oil content in optimal conditions.

In this study, we used *C. reinhardtii* 21 gr (wild type) and CC503 (wall-less) strains as a green alga model system and three *Chlorella* strains under photoautotrophic and nitrogen deprivation conditions. The total lipid and TAG contents were compared using two different lipid analysis methods, HPLC–ELSD and GC/MS with TLC. *C. sorokiniana* UTEX 1230, CS-01 and *C. kessleri* UTEX 2229 were selected according to their phylogenetic relationship with *C. variabilis* NC64A [24], a model *Chlorella* that has been extensively characterized. Differences in biomass production among *Chlorella* species also served as a variable of interest in relationship to lipid production. The HPLC–ELSD method resolves major lipid classes and rivals similar accuracy of quantification of TAG levels in comparison to TLC/GC methods when evaluating the *Chlorella* strains as well as the *Chlamydomonas* strains. This method provides a rapid and simple means to detect and quantify algal neutral lipid classes and is an efficient screening tool for metabolic studies of algal lipid accumulation.

## Materials and Methods

### Materials and Growth Conditions

*Chlamydomonas. reinhardtii* 21 gr (wild type) and CC503 (wall-less) were provided by Dr. K. Horken (University of Nebraska-Lincoln). *C. kessleri* UTEX 2229 and *C. sorokiniana* UTEX 1230 were obtained from The Culture Collection of Algae (UTEX) at the University of Texas at Austin. *C. sorokiniana* CS-01 was provided by Dr. M. Wan (Johns Hopkins University). *Chlamydomonas* strains were maintained in 100 mL TAP media [25] and *Chlorella* strains were plated on Bold’s Basal Medium (BBM, [26]) agar plates containing 10 μg/mL tetracycline and 100 μg/mL ampicillin. Liquid cultures of *Chlorella* were initiated by inoculation of a single isolated colony into 5 mL of BBM. Both species were grown for 14 days at 300 rpm under continuous illumination (160 μmol m^−2 ^s^−1^) at 25 °C.

Cultures were scaled up to 250-mL flasks and the cells were collected at the stationary phase by centrifugation at 5,000*g* for 10 min. Each cell pellet was transferred into 3 L of TAP media for *Chlamydomonas* strains and BBM media for *Chlorella* strains in a 3-L photo-bioreactor under continuous illumination at 200 μmol m^−2 ^s^−1^ in a greenhouse environment at 26 ± 2 °C. The 3-L photo-bioreactors were aerated by an air-pump (Whitewater, MTL66) with a flow rate at 1 psi 2.3 cfm in addition to a spinning turbine impeller in the photo-bioreactor set at 180 rpm. After 5 days at the stationary phase of *Chlamydomonas* strains and 12 days of *Chlorella* strains, cultures were divided into two 1.5-L aliquots that were centrifuged at 5,000*g* for 5 min and transferred into either 3 L TAP media or 3 L TAP media without a nitrogen source for *Chlamydomonas* strains or either 3 L BBM media or 3 L BBM media without a nitrogen source for *Chlorella* strains. The cultures were maintained for 7 days under the same conditions as above. The algae cells were collected by centrifugation at 5,000*g* for 5 min. The supernatant was discarded and pellets were stored at −80 °C and lyophilized overnight. The dried algae samples were used for lipid analyses.

*Chlorella* liquid cultures equal to and >300 mL were grown in photo-bioreactors under continuous illumination at 200 μmol m^−2^ s^−1^ in a greenhouse environment at 26 ± 2 °C. After incubation for 14 days, 15 mL cultures were transferred to 300 mL BBM in 500-mL aerated photo-bioreactors. Exponential-phase cells were transferred after 5 days to 3 L BBM in 3-L aerated photo-bioreactors and grown for an additional 7 days. Aeration flow for the 500-mL and 3-L photo-bioreactors were maintained by an air-pump (Whitewater, MTL66) with a flow rate of 1 psi 2.3 cfm in addition to agitation by spinning turbine impellers (180 rpm). The 3 L cultures were again scaled-up and the cells at 4 × 10^6^ cell/mL of each strain were transferred to 80 L BBM into 170-L open aquarium tanks with two Power Head water pumps (1,500 L/h; PETCO King 225) arranged in diagonal corners. Cell growth was measured by monitoring the absorbance at 530 nm and using a hemocytometer. Cells were harvested following a 10-day incubation period during the early stages of stationary phase. 2-L aliquots of cell samples from the aquarium tanks were collected by centrifugation at 5,000*g* for 5 min. Samples were stored at −20 °C until used for lipid analysis.

### Extraction of Lipids

The dried algae pellets (50, 100, 200 mg) were homogenized using a mortar and pestle with liquid nitrogen and used for the comparison of both TLC/GC and HPLC–ELSD methods. Lipid extraction was performed by a modification of the method described by Bligh and Dyer [27]. Two milliliters of chloroform/methanol (1:2 v/v) containing 0.01 % butyl hydroxyl toluene was added to the ground cells and pooled in a glass tube and repeated three times. Then, 500 μg of tripentadecanoin (15:0 TAG, Nu-Check Prep, MN, USA) was added as an internal standard for quantification of the FAME analysis. The mixture was vortexed at room temperature for 30 min. 2 mL of chloroform and 4 mL of water were added and vortexed. After centrifugation at 1,500*g* for 5 min, the chloroform phase was collected and the aqueous phase was re-extracted with 5 mL chloroform. This was repeated three times. The pooled chloroform phases were evaporated to dryness under a stream of nitrogen. The total lipid content was determined gravimetrically.

### Chromatographic Methods

**Fig. 1:**
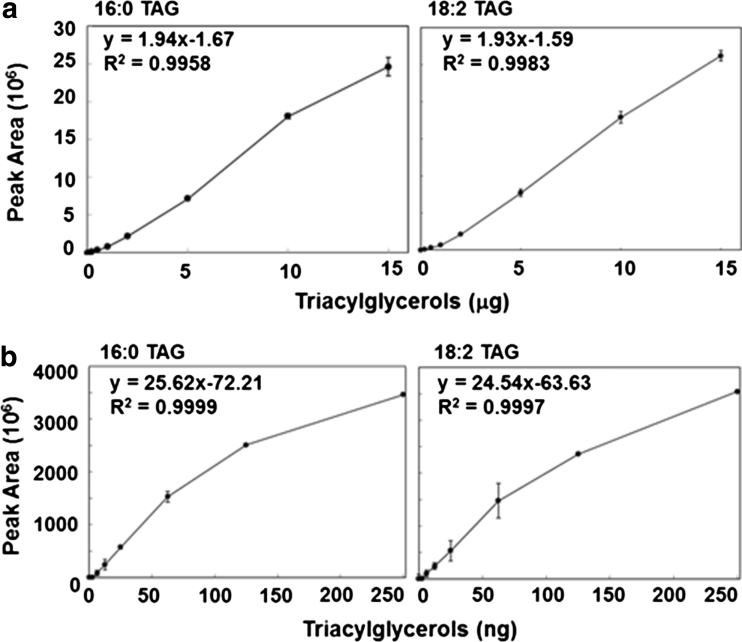
Comparison of calibration curves and their equations of trihexadecanoin (16:0 TAG) and trioctadecadienoin (18:2 TAG) between HPLC–ELSD (**a**) and GC–MS (**b**)

The separation of lipids by system gold HPLC (Beckman Coulter, CA, USA) with ELSD (Shimadzu, MD, USA) was performed in the gradient mobile phase (Table 1). The Luna 3-μm silica column (100 × 4.60 mm, Phenomenex, CA, USA) was connected with the guard column (silica 4 × 3.0 mm, Phenomenex, CA, USA), which incubated at 50 °C in the column oven. ELSD settings included gain 5, at 30 °C and 350 kPa for nitrogen pressure. The flow rate was 1 mL/min and 20 μL of the samples were injected. The solvent system used hexane based solvent A (hexane/isopropanol/acetic acid, 98.9:1:0.1 v/v/v, all HPLC grade, Fisher Scientific, PA, USA) and isopropanol based solvent B (isopropanol/acetic acid, 99.9:0.1 v/v/v, all HPLC grade, Fisher Scientific, PA, USA). After a 7-min isocratic run with solvent A for elution of neutral lipids, solvent B increased to 95 % for a 1-min wash and then the column was equilibrated by 100 % of solvent A for 6 min (Table 1). Seven TAG including tripentadecanoin, trihexadecanoin (palmitin), triheptadecanoin (margarin), trioctadecanoin (stearin), trioctadecenoin (vaccenin), triocadecadienoin (linolein) and trioctadecatrienoin (alpha-linolenin) (15:0, 16:0, 17:0, 18:0, 18:1, 18:2 and 18:3, Nu-Check Prep MN, USA) in hexane were applied for the reproducibility of peak area and retention times at 1.0 and 5.0 μg (Table 2) and measured from the ranges of 0–15 μg for the calibration curve (Fig. 1a). Then, 1 mL of hexane was added to the dried total lipid samples. SPE silica column (Supelclean LC-Si SPE Tubes 3 mL, SUPELCO Analytical, PA, USA) was equilibrated by hexane and 200 μL of the lipid mixture was added to the equilibrated SPE column. 6 mL of hexane was added and the elution was collected gravitationally after removal of chlorophyll and carotenoid fractions. Samples were stored at −20 °C. 10 mg/mL of TAG, DAG and palmitic acid (TAG, DAG and palmitic acid, Nu-Check Prep MN, USA) were dissolved in hexane. Then, 10 mg/mL of phosphaphatidylcholine (PtdCho) and phosphoethanolamine (PE), 1 mg/mL of MGDG, DGDG and dipalmitoyl glycero trimethyl homoserine (DGTS) (PtdCho, PE, MGDG, DGDG and DGTS, Avanti Polar Lipids, Inc., AL, USA) were dissolved in chloroform/methanol (8:2, v/v) for the authentic standards in Figs. 3a, 5a, 7a. Carotenoids were separated from *C. reinhardtii* by TLC (silica plate, Sigma-Aldrich) in heptane/diethyl ether/acetic acid (70:30:1, v/v/v, all Fisher Scientific). The yellow carotenoids were scraped and eluted in 3 mL of hexane. The eluted carotenoids were used for the authentic standards in Figs. 3a, 5a, 7a. The percentages of DW were calculated based on the equations from the TAG calibration curves and all the presented data incorporate averages ± standard deviations of three technical replicates.

**Table 1:** HPLC gradient condition of mobile phase

Time (min)	Percentage of solvent		Flow-rate (mL/min)
	A	B	
0	100	0	1
7	100	0	1
8	5	95	1
9	100	0	1
15	100	0	1

*A* 98.9 % hexane, 1.0 % isopropanol, 0.1 % acetic acid, *B* 99.9 % isopropanol, 0.1 % acetic acid

**Table 2:** Reproducibility of peak areas and retention times of TAG standards injected 12 times

TAG	Conc. (μg)	Retention time (min)			Peak area		
		Ave.	SD	% RSD	Ave.	SD	% RSD
15:0	1.0	1.92	0.03	0.9	875,942	46,138	5.3
	5.0	1.95	0.01	0.4	9,278,987	281,635	3.0
16:0	1.0	1.94	0.02	0.8	805,907	57,103	7.1
	5.0	1.97	0.02	0.8	8,580,230	277,905	3.2
17:0	1.0	1.96	0.02	0.6	706,495	53,524	7.0
	5.0	1.95	0.03	1.0	7,517,341	277,102	3.7
18:0	1.0	1.91	0.01	0.4	772,662	54,400	7.0
	5.0	1.91	0.02	0.7	4,764,890	275,968	5.8
18:1	1.0	1.92	0.03	1.1	814,658	40,017	4.9
	5.0	1.92	0.01	0.5	9,152,411	424,981	4.6
18:2	1.0	1.93	0.03	0.7	918,516	44,708	4.9
	5.0	1.94	0.02	0.6	8,625,403	356,563	4.1
18:3	1.0	1.92	0.02	0.7	928,705	59,874	6.4
	5.0	1.92	0.02	0.9	9,576,351	527,472	5.5

*Conc.* concentration, *Ave.* average, *SD* standard deviation, *RSD* ratio of SD and Ave

TLC for neutral lipid was separated by heptane/diethyl ether/acetic acid (70:30:1, v/v/v) and TAG were co-migrated at the same position as the standard TAG from soy beans stained by iodine stream and scraped for FAME (fatty acid methyl esters) analysis. Trans-methylation to FAME was performed as previously described [3]. FAME were analyzed using an Agilent 6890 Series GC System with Agilent 5973 Network Mass Selective Detector (Agilent Technologies, DE, USA). Chromatography was carried out using a 200 m × 250 μm × 0.25 μm Varian GC Capillary column (Varian Inc., CA, USA). The GC inlet was held at 270 °C and 1 μL of the sample was injected in splitless mode. The oven temperature was programmed from 130 °C (10 min hold) to 160 °C (7 min hold), from 160 to 190 °C (7 min hold), from 190 to 220 °C (22 min hold) and from 220 to 250 °C (17 min hold) at a rate of 10 °C/min for each step with helium as the carrier gas. The total analysis time was 75 min. The GC/MS was carried out using 70 eV EI and the data was evaluated with the total ion count. The total lipid content was calculated based on the 15:0 TAG internal standard and the ration of peak area of the total FAME composition sum and 15:0 FAME was applied to obtain the lipid content percentages in DW. All data include averages ± standard deviations of three technical replicates.

## Results and Discussion

### Development of Triacylglycerol Detection and Quantification by HPLC–ELSD Methods

Gas chromatographic analysis combined with purification of lipid classes by silica TLC or column chromatography is commonly used for TAG analyses. However, this approach is time-consuming and labor intensive and cannot be used to analyze large numbers of samples efficiently. In order to develop a rapid method for measuring TAG accumulation in *C. reinhardtii* 21 gr (wild type) and CC503 (wall-less) under conditions of nitrogen depletion, total neutral lipids were extracted and separated by HPLC–ELSD. The separation was performed by a silica column (Luna 3 μm silica 100 × 4.6 mm) with a solvent system consisting of a non-polar solvent A (hexane/isopropanol/acetic acid, 98.9:1:0.1 v/v/v,) and a more polar solvent B (isopropanol/acetic acid, 99.9:0.1 v/v,), allowing for resolution of the major lipid classes (carotenoids, triacylglycerides, diacylglycerides, free fatty acids, phospholipids and glycolipids). Seven TAG including tripentadecanoin, trihexadecanoin (palmitin), triheptadecanoin (margarin), trioctadecanoin (stearin), trioctadecenoin (vaccenin), triocadecadienoin (linolein) and trioctadecatrienoin (alpha-linolenin) (15:0, 16:0, 17:0, 18:0, 18:1, 18:2 and 18:3 TAG) were selected as standards and reproducibility of retention times and peak areas are compared in Table 2. Two different concentrations (1.0 and 5.0 μg) of each standard were injected 12 times. All of the retention times were averaged at 1.9 min. The percentage of the ratio of standard deviation and the average (%RSD) retention times were 0.4–1.1 in all standards at both concentrations. The averages of peak areas were 0.7–0.9 × 10^6^ at 1.0 μg and 8.2–9.6 × 10^6^ at 5.0 μg with outliers 7.5 × 10^6^ and 4.8 × 10^6^ as 17:0 and 18:0 TAG, respectively. The % RSD of peak areas at 1 and 5 μg were at 4.9–7.1 and 3.0–5.8 respectively that indicated the stability of TAG in this HPLC system (Table 2).

### Comparison of Calibration Curves Measured by HPLC–ELSD and GC/MS

GC analyses in *Chlamydomonas* in addition to other algal species confirmed FAME 16:0 and 18:2 as primary fatty acids in lipid profiles [3, 10], therefore trihexadecanoin (16:0 TAG) and trioctadecenoin (18:2 TAG) were selected as authentic standards and their calibration curves are shown in Fig. 1. The concentration ranges of the calibration curves for both TAG by HPLC–ELSD were from 0.5 to 15 μg. Following the 15 μg peaks, saturation of the authentic standard created a plateau in the standard curve. The equations from the calibration curves were

1 $$ y = 1.94x - 1.67 $$

for trihexadecanoin and

2 $$ y = 1.93 \, x - 1.59 $$

for trioctadienoin based on the peak area points at 1, 2.5, 5 and 10 μg by HPLC–ELSD. In contrast, both TAG were measured at the mass ranges from 5 to 250 ng by GC/MS. The calibration curves exhibited a linear trend at concentrations of 5, 10, 25, and 50 ng and presented a plateau fashion following 100 ng. The equations from the linear ranges of the calibration curves by GC/MS were

3 $$ y = 25.62x - 72.2 $$

for trihexadecanoin and

4 $$ y = 24.54 \, x - 63.63 $$

for trioctadienoin. The equations show no significant difference between trihexadecanoin and trioctadienoin by both HPLC–ELSD (Eqs. 1, 2) and GC/MS (Eqs. 3, 4), however the detection level of 1–10 μg TAG by HPLC–ELSD is higher than that of 5–50 ng by GC/MS. This indicates the sensitivity of GC is 1,000 times higher than using HPLC–ELSD.

### FAME Analysis by TLC/GC and Separation of Different Lipid Classes by HPLC–ELSD in *C. reinhardtii*

**Fig. 2:**
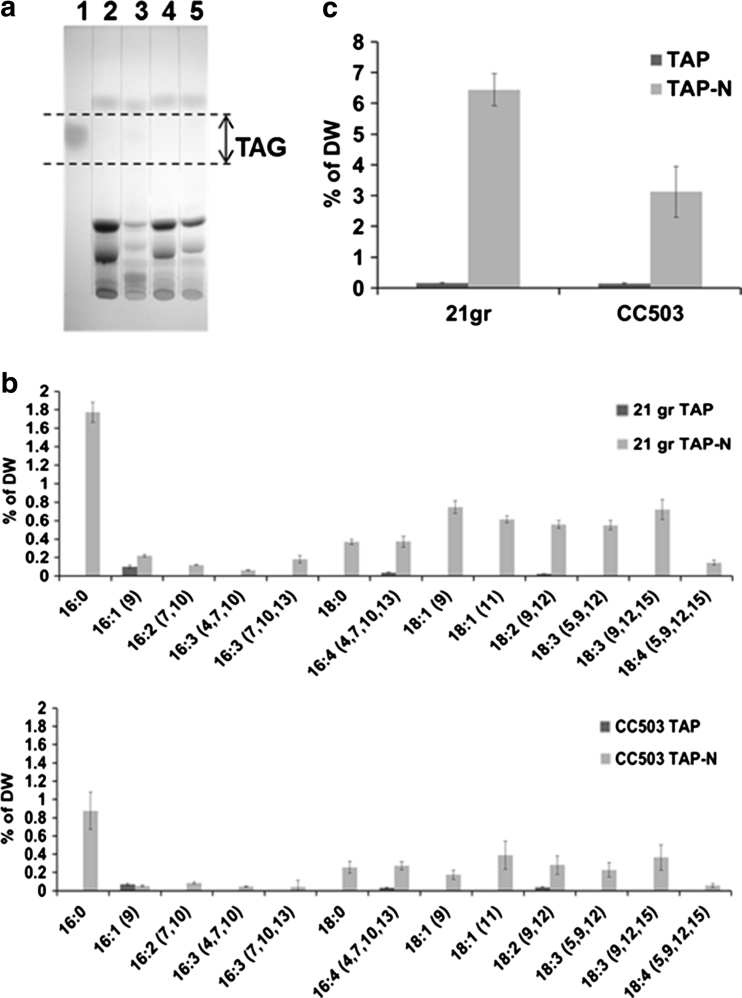
TAG analysis by TLC/GC method in *C. reinhardtii* 21 gr and CC503 under TAP and TAP-N conditions. **a** TAG separation by TLC.* Lane 1*, soy extraction stained by iodine stream; 2, 21 gr in TAP; 3, 21 gr on TAP-N; 4, CC503 in TAP; 5, CC503 in TAP-N. *Arrow region* shows the TAG part to be scraped. **b** TAG lipid compositions of 21 gr and CC503 under TAP and TAP-N conditions. **c** TAG contents of 21 gr and CC503 under TAP and TAP-N conditions quantified by GC/MS. *Error bar* *n* = 3 technical replicates from one 3-L bioreactor culture. Ave. of % DW ± standard deviation

Two *C. reinhardtii* strains, 21 gr (wild type) and CC503 (wall-less), were grown in TAP and TAP media without a nitrogen source. TAG levels were compared by TLC/GC and HPLC–ELSD. TAG contents were first quantified by the conventional TLC/GC method. After lipid extraction, TAG in the crude lipid extract was separated by TLC and TAG on the TLC was scraped at the same position as the co-migrated soy TAG (Fig. 2a). The scraped TAG was eluted and trans-esterified to FAME and the FAME were analyzed as lipid compositions by GC/MS (Fig. 2b). The total lipid and TAG compositions of 21 gr and CC503 under nitrogen depletion were analyzed as the same types of compositions; 16:0 16:1–16:4 and 18:0–18:4 (carbon number: double bond number, the major fatty acid). The fatty acid profile shown in Fig. 2b is consistent with that reported previously in other *C. reinhardtii* strains [3, 10]. After FAME separation by GC/MS, the concentration of FAME was calculated based on the internal standard and total TAG contents were estimated as the sum of the FAME and expressed as the percentage of the DW (Fig. 2b, c). The TAG contents of 21 gr and CC503 under nitrogen deprivation were 6 and 3 % respectively but below 1 % in regular TAP media (Fig. 2c). The 3 % of CC503 TAG content under nitrogen depletion (biomass 5.28 mg/L) at day 7 and was lower than the 6 % TAG content of 21 gr (biomass 43.0 mg/L) due to biomass differences, which might indicate that the wall-less strain CC503 produces a lower oil production in comparison to the wild type 21 gr.

**Fig. 3:**
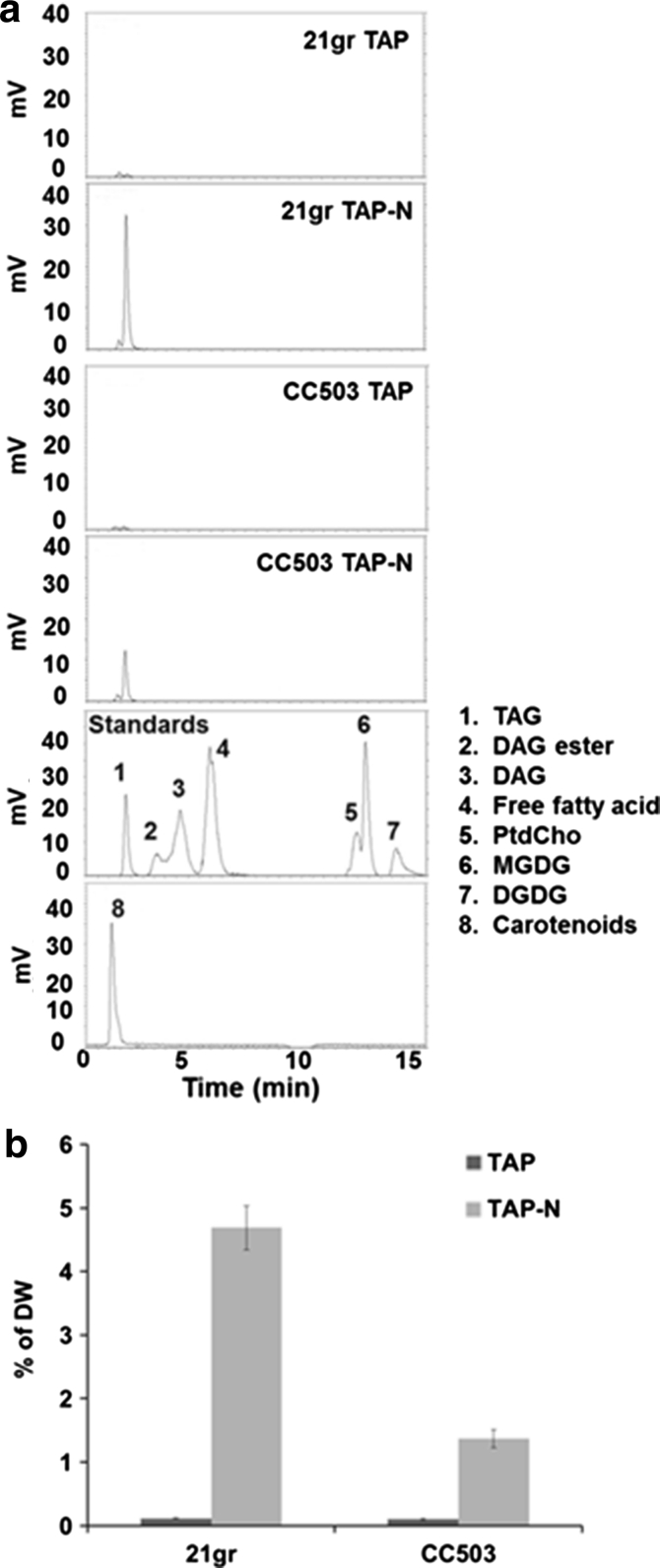
**a** Separation of TAG from other neutral-, polar-lipids and glycolipids in *C. reinhardtii* 21 gr and CC503 under TAP and TAP-N conditions and authentic standards by HPLC–ELSD.* 1* triacylglycerols (TAG, 1 μg),* 2* diacylglycerol (DAG) ester* 3* diacylglycerol (DAG, 4 μg),* 4* free fatty acids (palmitic acid 2 μg),* 5* phosphaphatidylcholine (PtdCho, 20 μg),* 6* monogalactosyldiacylglycerol (MGDG, 20 μg),* 7* digalactosyldiacylglycerol (DGDG, 40 μg)* 8* carotenoids extracted from *C. reinhardtii*. **b** TAG contents of 21 gr and CC503 under TAP and TAP-N conditions estimated by HPLC–ELSD. *Error bar* *n* = 3 technical replicates from one 3-L bioreactor culture. Ave. of % DW ± standard deviation

The separation of TAG from carotenoids, DAG, phospholipids, galactolipids and phospholipid in *C. reinhardtii* 21 gr and CC503 under nitrogen depletion is shown in Fig. 3a. In the chromatograph of authentic standards and carotenoids extracted from *C. reinhardtii*, carotenoids, TAG, DAG and free fatty acids were eluted at the non-polar phase for 7 min, followed by a 1-min wash phase then PtdCho, MGDG and DGDG were eluted by isopropanol for another 7 min. The retention times of carotenoids (8), TAG (1), diacylglycerol ester (DAG ester, 2), DAG (3) and palmitic acid as free fatty acids (4) were eluted at 1.6, 1.9, 3.2, 4.1 and 5.5 min respectively and the retention times of phosphatidylcholine (PtdCho, 5), monogalactosyldiacylglycerol (MGDG, 6) and digalactosyldiacylglycerol (DGDG, 7) were at 12.0, 12.3 and 13.7 min respectively. Because the solvent system started from a non-polar solvent in this HPLC system and considering the solubility of the polar lipids in a non-polar solvent, detection of the phospholipid and glycolipid standards required 10 times higher concentrations than those of neutral lipids. Some phospholipids and glycolipids such as PE and DGTS could not be detected in this system. Sulfolipid SQDG has less than or similar solubility to DGTS and therefore also did not display a peak in this system [28, 29]. After the respective modification of [9, 30], detection of the different classes of lipids and separation of TAG were able to be improved in this system. The separation of lipids was characteristic in *C. reinhardtii* 21 gr and CC503 under nitrogen depletion. Considering the difference in total lipids between the gravimeter and FAME was more than 50 % in *C. reinhardtii*, the crude lipid extract was dissolved in non-polar hexane and filtered using a SPE column to remove the high amount of non-fatty acid types of lipids and served to protect the HPLC column. In both 21 gr and CC503 under nitrogen depletion, carotenoids as a minor lipid and TAG as a major lipid were detected at 1.6 and 1.9 min respectively. DAG ester, DAG and free fatty acids in the non-polar elution phase and PtdCho, MGDG and DGDG after isopropanol wash phase were not detected in this system (Fig. 3a). In contrast to the lipid profile by TLC (Fig. 2a), the profile by HPLC–ELSD was simpler (Fig. 3a). This is because the non-fatty acid types of lipids including glycolipids and phospholipids were removed by an SPE filter from the crude lipid extract and also the non-polar solvent was used for the running solvent in HPLC and polar lipid classes were less sensitive detection due to their reduced solubility compared to non-polar lipid classes, e.g. TAG, DAG and free fatty acids (1–4 μg) vs. PtdCho, MGDG and DGDG (20–40 μg). Traces of carotenoids and TAG were detected at 1.6 and 1.9 min respectively in both strains in TAP media. Considering 16:0 TAG demonstrated high reproducibility and a stable calibration curve (Table 2; Fig. 1) in combination with the major fatty acid in *C. reinhardtii*, palmitic acid (16:0), Eq. 1 from the calibration curves of trihexadecanoin (16:0 TAG) were applied to the TAG quantification of the samples from *C. reinhardtii* 21 gr and CC503 under nitrogen depletion. TAG content of 21 gr was 6 % and CC503 was 3 % under nitrogen depletion but below 1 % in TAP media. This indicates the values are as consistent as those estimated by the TLC/GC methods.

### Comparison of Lipid Contents by TLC/GC Methods and HPLC–ELSD in Three *Chlorella* Strains Under BBM and BBM Without Nitrogen (BBM-N)

Using a gravimeter and other different quantification methods, total lipid content in *C. sorokiniana* was reported to be over a wide range from 3 to 52 %. Lipids can be extracted by various methods such as Bligh and Dyer [27], Soxhlet with chloroform/methanol [31], direct trans-methylation [12, 32] or using an automated extraction system [33]. After the extraction, extracted lipids were analyzed by either gravimeter, gas chromatography or spectrophotometer and then quantified by total FAME based on dry weight (%DW), relative percentages from total FAME estimation, lipid productivity (mg/L/day) and lipid per cell (g/cell) [18, 31–37] .

**Fig. 4:**
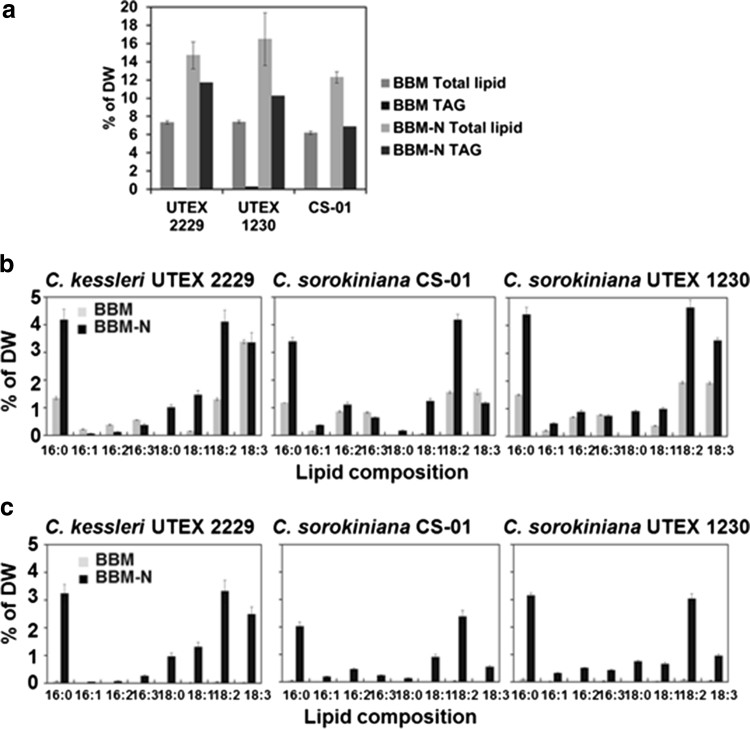
Contents and compositions of FAME total lipids and FAME triacylglycerols (TAG) of dried *Chlorella* by GC/MS under BBM and BBM in absence of nitrogen source (BBM-N). **a** Contents of FAME total lipids and TAG in *C. kessleri* UTEX 2229, *C. sorokiniana* CS-01 and UTEX 1230. **b** Compositions of FAME total lipids in *C. kessleri* UTEX 2229, *C. sorokiniana* CS-01 and UTEX 1230. **c** FAME TAG compositions in *C. kessleri* UTEX 2229, *C. sorokiniana* CS-01 and UTEX 1230. *Error bar* *n* = 3 technical replicates from one 3 L bioreactor culture. Ave. of % DW ± standard deviation

To obtain a rapid and accurate TAG content in *Chlorella*, the method using HPLC–ELSD was compared with the conventional TLC/GC method. Total lipid and TAG levels in *C. kessleri* UTEX 2229, *C. sorokiniana* CS-01 and UTEX 1230 were determined by the TLC/GC method under BBM and BBM-N growth conditions (Fig. 4). Figure. 4a shows that FAME total lipid content ranged from 12 to 17 % DW under BBM-N in all three *Chlorella* species, while 6–8 % DW under BBM. TAG accumulated 7–12 % DW under BBM-N, while below 0.3 % DW in BBM. Fatty acid composition of total lipids and TAG in the *Chlorella* species were compared in BBM and BBM-N and are shown in Fig. 4b, c. The fatty acids were detected at 16:0, 16:1, 16:2, 18:0, 18:1, 18:2 and 18:3 in both FAME total lipids and TAG, which are consistent with previous studies on the major fatty acids found in other *Chlorella* species such as *C. zofingiensis* [4], *C. vulgaris* [1, 38], *C. sorokiniana* [1, 18] and *C. protothecoides* [39].

The levels of each fatty acid differed under BBM and BBM-N. The ranges of FAME compositions in the total lipids were 0.2–4.5 % DW under BBM-N while 0.2–3 % under BBM in all *Chlorella* strains. The major fatty acids detected in the *Chlorella* strains were palmitic acid (16:0), linoleic acid (18:2) and linolenic acid (18:3). The respective ratio of fatty acids in *C. kessleri* UTEX 2229 measured 28, 28 and 23 % total lipids, *C. sorokiniana* CS-01 measured 28, 34 and 10 % total lipids and *C. sorokiniana* UTEX 1230 measured 27, 28 and 21 % total lipids under BBM-N conditions. The ratio of major fatty acids in *C. kessleri* UTEX 2229 measured 18, 18 and 46 % total lipids, *C. sorokiniana* CS-01 measured 19, 25 and 23 % total lipids and *C. sorokiniana* UTEX 1230 measured 20, 26 and 26 % total lipids under BBM. The levels of 16:0 and 18:2 of FAME total lipids under BBM-N in all *Chlorella* strains increased 2–3 times higher than those of BBM and the content of 18:3 of FAME total lipids under BBM-N in *C. sorokiniana* UTEX 1230 increased almost two times more than that in BBM condition. This might indicate the involvement of high palmitic acid production in plastid and also higher activity of fatty acid desaturase in the endoplasmic reticulum or plastid observed in plants [40, 41]. Figure 4c shows the fatty acid composition of TAG by the TLC/GC method. The compositions of all *Chlorella* strains under BBM-N showed the same pattern as the total lipid compositions and the TAG content was within the range of 0.01–4 % DW.

**Fig. 5:**
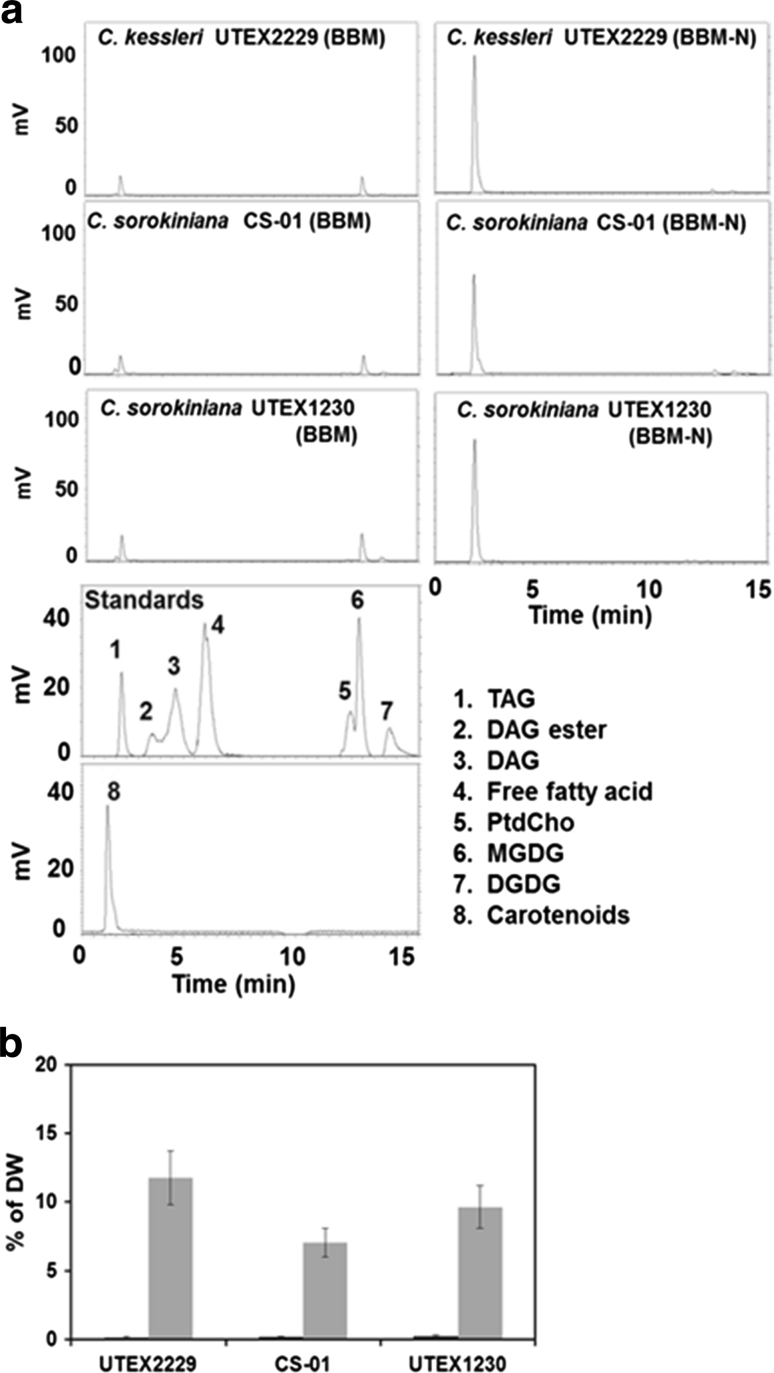
Comparison of TAG detection in *C. kessleri* UTEX 2229, *C. sorokiniana* CS-01, *C. sorokiniana* UTEX 1230 under BBM and BBM-N (**a**). Separation of neutral lipid from polar lipid and glycolipid, and authentic standards by HPLC–ELSD. *1* triacylglycerols (TAG, 1 μg), *2* diacylglycerol (DAG) ester *3* diacylglycerol (DAG, 4 μg), *4* free fatty acids (palmitic acid 2 μg), *5* phosphaphatidylcholine (PtdCho, 20 μg), *6* monogalactosyldiacylglycerol (MGDG, 20 μg), *7* digalactosyldiacylglycerol (DGDG, 40 μg), *8* carotenoids extracted from *C. reinhardtii*. (**b**) TAG contents of *C. kessleri* UTEX 2229, *C. sorokiniana* CS-01 and UTEX 1230 under BBM and BBM-N by HPLC–ELSD. *Error bar* *n* = 3 technical replicates from one 3-L bioreactor culture. Ave. of % DW ± standard deviation

The separation of lipids was characteristic in the three *Chlorella* strains under BBM and BBM-N by HPLC–ELSD (Fig. 5a). Under BBM conditions TAG and a trace of carotenoids were detected at 1.9 and 1.7 min respectively in the non-polar elution phase while MGDG and traces of DGDG were measured at 13 and 13.8 min respectively after the isopropanol wash phase in all *Chlorella* strains. Under BBM-N conditions, the dominant TAG peak was detected at 1.9 min and traces of unknown peaks were detected, which might indicate that the flow toward the TAG biosynthesis pathway is dominant under BBM-N condition. The equations from the calibration curves of trihexadecanoin (16:0 TAG, Eq. 1) were applied to the TAG quantification of the samples from *C. kessleri* UTEX 2229, *C. sorokiniana* CS-01 and UTEX 1230 under BBM and BBM-N. The levels of *Chlorella* TAG detected by HPLC–ELSD under BBM and BBM-N are shown in Fig. 5b. The TAG contents in *C. kessleri* UTEX 2229, *C. sorokiniana* CS-01 and UTEX 1230 were 12, 7 and 10 % DW, respectively under BBM-N, while all the *Chlorella* strains under BBM was under 0.2 % DW.

### FAME Analysis by TLC/GC and Separation of Different Lipid Classes by HPLC–ELSD in *Chlorella* Strains

**Fig. 6:**
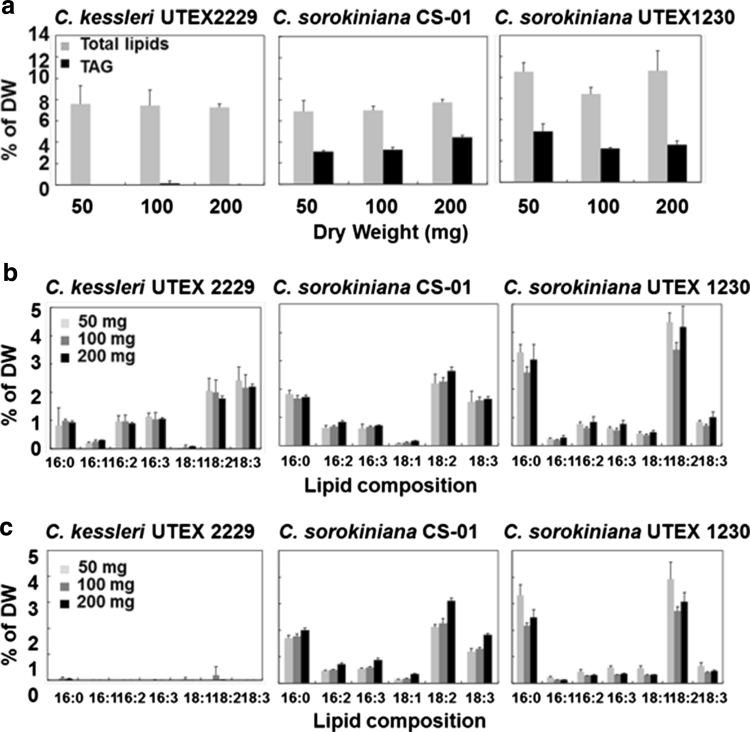
Contents and compositions of FAME total lipids and FAME triacylglycerols (TAG) at 50, 100 and 200 mg of dried *Chlorella* by GC/MS at the late stationary phase. **a** Contents of FAME total lipids and TAG in *C. kessleri* UTEX 2229, *C. sorokiniana* CS-01 and UTEX 1230. **b** Compositions of FAME total lipids in *C. kessleri* UTEX 2229, *C. sorokiniana* CS-01 and UTEX 1230. **c** FAME TAG compositions in *C. kessleri* UTEX 2229, *C. sorokiniana* CS-01 and UTEX 1230. *Error bar* *n* = 3 technical replicates from one 80 L aquarium culture. Ave. of % DW ± standard deviation

To assess accuracy of lipid analysis using the HPLC–ELSD method, the lipid contents were compared not only under nitrogen deprivation conditions but also in different amounts of DW. The comparison of the lipid analysis by the TLC/GC and HPLC–ELSD methods were performed with *C. kessleri* UTEX 2229, *C. sorokiniana* CS-01 and UTEX 1230. Total lipid and TAG levels in the three *Chlorella* strains were first determined by the TLC/GC method (Fig. 6). Figure 6a shows the range of FAME total lipid content from 7 to 11 % DW in all three *Chlorella* strains and TAG accumulation from 4 to 6 % DW in *C. sorokiniana* CS-01 and UTEX 1230 under late stationary phase. In contrast, TAG accumulation in *C. kessleri* UTEX 2229 was hardly detectable. The fatty acid composition of the total lipids and TAG in *C. kessleri* UTEX 2229, *C. sorokiniana* CS-01 and UTEX 1230 are shown in Fig. 6b, c. The fatty acids detected were 16:0, 16:1, 16:2, 18:0, 18:1, 18:2 and 18:3 both in the FAME total lipids and TAG. The levels of each fatty acid differed in the three *Chlorella* strains. The ranges of FAME compositions in total lipids were 0.2–3 % DW in both *C. kessleri* UTEX 2229 and *C. sorokiniana* CS-01 while slightly higher in *C. sorokiniana* UTEX 1230, 0.3–5 % DW. The major fatty acids detected in the *Chlorella* strains were palmitic acid (16:0), linoleic acid (18:2) and linolenic acid (18:3). Respective major fatty acids in *C. kessleri* UTEX 2229 were measured at 12, 26 and 30 % total lipids, *C. sorokiniana* CS-01 measured 24, 33 and 22 % total lipids, and *C. sorokiniana* UTEX 1230 measured 30, 40 and 9 % total lipids. Figure 6c shows the fatty acid composition and content of purified TAG after separation by TLC and analysis and quantification by GC analyses. The compositions of *C. sorokiniana* CS-01 and UTEX 1230 showed the same pattern as the total lipid compositions and the TAG content was within the range of 0.3–4 % DW, however in *C. kessleri* UTEX 2229 TAG was nearly undetectable. Although total lipids in *C. kessleri* UTEX 2229 accumulated to the same level as *C. sorokiniana* CS-01 and UTEX 1230, it is unclear whether the failure to detect TAG in *C. kessleri* UTEX 2229 was due to the absence of TAG synthesis and accumulation of TAG or rapid degradation of TAG. While the DW of *Chlorella* species differed from 50, 100 and 200 mg, the percentages of FAME total lipids and TAG per DW, excluding TAG of *C. kessleri* UTEX 2229, demonstrated consistency at 8–12 and 3–5 %, respectively using this method (Fig. 6a–c).

**Fig. 7:**
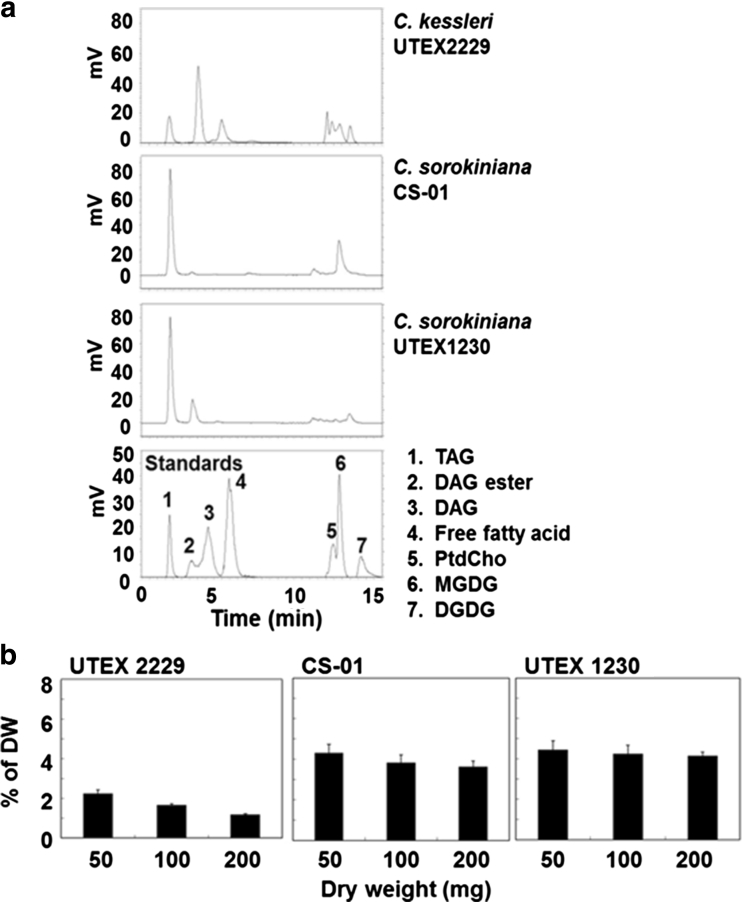
Comparison of TAG detection and contents in *C. kessleri* UTEX 2229, *C. sorokiniana* CS-01, *C. sorokiniana* UTEX 1230. **a** Separation of neutral lipid from polar lipid and glycolipid, and authentic standards by HPLC–ELSD. *1* triacylglycerols (TAG, 1 μg), *2* diacylglycerol (DAG) ester *3* diacylglycerol (DAG, 4 μg), *4* free fatty acids (palmitic acid 2 μg), *5* phosphaphatidylcholine (PtdCho, 20 μg), *6* monogalactosyldiacylglycerol (MGDG, 20 μg), *7* digalactosyldiacylglycerol (DGDG, 40 μg), *8* carotenoids extracted from *C. reinhardtii.* **b** TAG contents of *C. kessleri* UTEX 2229, *C. sorokiniana* CS-01 and UTEX 1230 at 50, 100 and 200 mg by HPLC–ELSD. *Error bar* *n* = 3 technical replicates from one 80-L aquarium culture. Ave. of % DW ± standard deviation

The separation of lipids was characteristic in *Chlorella* by HPLC–ELSD (Fig. 7a). In *C. kessleri* UTEX 2229 TAG, DAG and free fatty acids were detected after 1.8, 3.7 and 5.1 min, respectively in the non-polar elution phase while PtdCho, MGDG and DGDG were measured after 11.9, 12.4 and 13.2 min, respectively after the isopropanol wash phase. The CS-01 TAG peak was detected at 1.8 min and MGDG was detected at 12.3 min. In *C. sorokiniana* UTEX 1230 TAG and DAG were detected at 1.8 and 2.8 min, respectively with other unknown trace peaks. The equation from the calibration curves for trihexadecanoin (Eq. 1) was applied to the TAG quantification of the samples from *C. kessleri* UTEX 2229, *C. sorokiniana* CS-01 and UTEX 1230. The levels of *Chlorella* TAG detected by HPLC–ELSD are shown in Fig. 7b. The levels of TAG in *C. kessleri* UTEX 2229 were <2.5 % DW which is below the detection limit by GC/MS analysis. In *C. sorokiniana* CS-01 and UTEX 1230 levels of TAG were 5 % DW and were consistent between different amounts of DW (50, 100 and 200 mg).

The HPLC–ELSD system was able to detect TAG at a level which was below detection by GC analysis and also allowed us to separate not only TAG but also other lipids such as DAG, free fatty acids, PtdCho, MGDG and DGDG in a single 15 min HPLC run. Although the levels of total lipids in *C. kessleri* UTEX 2229 by GC/MS analysis were at the same levels as those of *C. sorokiniana* CS-01 and UTEX 1230, only the levels of TAG in *C. kessleri* UTEX 2229 were below detection (Fig. 6a). However, application of HPLC–ELSD to lipids of *C. kessleri* UTEX 2229 showed lower levels of TAG while a higher peak of DAG and free fatty acids in the same trail run (Fig. 7a). A previous study examining the lipid profile of *C. minutissima* grown under nitrogen starvation reported TAG and monoglyceride were the major lipid classes, and free fatty acid and DAG were reduced as assessed using HPLC–ELSD [23]. This might indicate the degradation of TAG or less activity of diacylglycerol acyl transferase that converts DAG to TAG [42, 43].

### Comparison of Total Lipid Content by Gravimeter and FAME Total Lipid and TAG Contents by TLC/GC and HPLC–ELSD Methods

Total lipid contents of the wild type strain 21 gr by gravimeter were 24 and 23 % and those of CC503 were 21 and 18 % of DW, while FAME total lipid contents of 21 gr were 10 and 14 % and those of CC503 were 7 and 8 % DW under TAP and TAP without nitrogen conditions, respectively (Table 3). This difference between total lipids by gravimeter and FAME total lipids indicates that the non-fatty acid type of lipids such as chlorophyll, carotenoids, tocopherols and sterols might represent 50 % of the total lipids in *C. reinhardtii* 21 gr and CC503. The FAME TAG contents of 21 gr and CC503 were measured at <1 % in TAP media and 6 % (wild type 21 gr) and 3 % DW (CC503) under nitrogen depletion by both TLC/GC and HPLC–ELSD methods. Under conditions of nitrogen depletion, TAG accumulated in *C. reinhardtii* 21 gr and CC503, and that the prominent TAG peak that is used to quantitate TAG accumulation by HPLC–ELSD represents primarily TAG and is not a mixture of other cell lipids (for example carotenoids, which would result in an overestimation of TAG content by HPLC–ELSD if co-eluting with the TAG peak).

**Table 3:** Comparison of total lipids, FAME total lipids and TAG quantification by gravimeter, HPLC–ELSD and GC/MS in *C. reinhardtii*

*C. reinhardtii*	Gravimeter	GC/MS		HPLC–ELSD
	Total lipids	FAME total lipids	FAME TAG	TAG
21 gr				
TAP	23.67 ± 0.37	9.96 ± 0.95	0.17 ± 0.02	0.11 ± 0.01
TAP-N	23.12 ± 0.38	13.88 ± 0.88	6.44^a^ ± 0.53	6.44^b^ ± 0.34
CC503				
TAP	20.95 ± 1.21	7.42 ± 0.14	0.14 ± 0.02	0.10 ± 0.02
TAP-N	17.63 ± 1.10	7.85 ± 0.27	3.12^c^ ± 0.08	3.15^d^ ± 0.18

Error bar: *n* = 3 technical replicates from one 3 L bioreactor culture. Ave. of % DW ± standard deviation

^a^ 54.59 (sum of peak area)/3.89 (15:0 peak area) × 500 μg (internal standard)/1,000 = 7.02 mg, 7.02 mg × 100/109 (mg DW) = 6.44 %

^b^ {4.38 (peak area) + 1.67/1.94} × 2,250 (dilution factor)/1,000 = 7.02 mg, 7.02 mg × 100/109 (mg DW) = 6.44 %

^c^ 24.03 (sum of peak area)/3.57 (15:0 peak area) × 500 μg (internal standard)/1,000 = 3.38 mg, 3.38 mg × 100/108 (mg DW) = 3.12 %

^d^ {1.26 (peak area) + 1.67/1.94} × 2,250 (dilution factor)/1,000 = 3.40 mg, 3.40 mg × 100/108 (mg DW) = 3.15 %

The quantification of total lipids and TAG contents by gravimeter, GC/MS and HPLC–ELSD in three *Chlorella* strains under BBM and BBM-N are shown in Table 4. Total lipid content under BBM-N measured by gravimeter ranged from 17 to 26 % DW while only 16–19 % DW under BBM in all the *Chlorella* strains. The FAME total lipid content determined by GC/MS was 12–16 % DW under BBM-N, while 6–7 % DW under BBM. The levels of TAG measured by GC/MS with TLC or HPLC–ELSD were similar at 12, 7 and 10 % DW in *C. kessleri* UTEX 2229, *C. sorokiniana* CS-01 and UTEX 1230 respectively, however under BBM, TAG levels ranged from 0.2 to 0.3 % DW in all the *Chlorella* strains which further indicates the consistency of the TAG content measured by the TLC/GC and HPLC–ELSD methods. In comparison to BBM, BBM-N conditions accumulated twice the contents of FAME total lipids and 2–3 times higher TAG levels in the late stationary phase in *C. sorokiniana* (Figs. 4a vs. 6a; Tables 4 vs. 5). Additionally, *Chlorella* in BBM-N accumulated 2–4 times higher than TAG contents in *C. reinhardtii* under nitrogen deprivation (Figs. 2c vs. 4a; Tables 3 vs. 4).

**Table 4:** Comparison of TAG quantification by HPLC–ELSD and GC/MS under BBM and BBM-N in *Chlorella*

*Chlorella*	Weight	GC/MS		HPLC–ELSD–LT
	Total lipids by a gravimeter	FAME total lipids	FAME TAG	TAG
*C. kessleri* UTEX 2229				
BBM	18.57 ± 0.35	7.35 ± 0.18	0.15 ± 0.02	0.17 ± 0.04
BBM-N	26.27 ± 2.02	14.70 ± 1.47	11.71 ± 1.30	11.77 ± 196
*C. sorokiniana* CS-01				
BBM	15.53 ± 0.41	6.20 ± 0.17	0.12 ± 0.04	0.19 ± 0.01
BBM-N	16.53 ± 1.04	12.28 ± 0.63	6.89 ± 0.62	7.06 ± 1.04
*C. sorokiniana* UTEX 1230				
BBM	18.78 ± 0.58	7.40 ± 0.16	0.28 ± 0.07	0.28 ± 0.08
BBM-N	23.72 ± 0.67	16.48 ± 2.89	10.24 ± 0.54	9.65 ± 1.53

Error bar: *n* = 3 technical replicates from one 3-L bioreactor culture). Ave. of  % DW ± standard deviation

*ND* not detected. Samples were measured as % DW

**Table 5:** Comparison of TAG quantification by HPLC–ELSD and GC/MS in *Chlorella*

*Chlorella*	Weight	GC/MS		HPLC–ELSD–LT
	Total lipids by a gravimeter	FAME total lipids	FAME TAG	TAG
*C. kessleri* UTEX 2229				
50 mg	18.73 ± 1.10	7.60 ± 1.72	ND	2.25 ± 0.19
100 mg	17.16 ± 0.54	7.47 ± 1.44	0.15 ± 0.22	1.66 ± 0.07
200 mg	17.87 ± 0.38	7.25 ± 0.32	0.04 ± 0.01	1.18 ± 0.06
*C. sorokiniana* CS-01				
50 mg	13.71 ± 1.32	6.89 ± 1.06	3.08 ± 0.13	4.13 ± 0.43
100 mg	14.29 ± 1.01	7.00 ± 0.20	3.29 ± 0.12	3.82 ± 0.38
200 mg	13.46 ± 1.62	7.76 ± 0.28	4.44 ± 0.19	3.62 ± 0.29
*C. sorokiniana* UTEX 1230				
50 mg	18.88 ± 0.90	10.56 ± 0.88	4.85 ± 0.73	4.46 ± 0.42
100 mg	14.05 ± 0.87	8.42 ± 0.67	3.20 ± 0.12	4.25 ± 0.41
200 mg	14.11 ± 1.09	10.66 ± 0.88	3.58 ± 0.36	4.14 ± 0.19

Error bar: *n* = 3 technical replicates from one 80 L aquarium culture). Ave. of % DW ± standard deviation)

*ND* not detected. Samples were measured as % DW

Table 5 shows the comparison of quantification of total lipids and TAG by gravimeter, GC/MS and HPLC–ELSD in the three *Chlorella* strains. Total lipid content measured by gravimeter ranged from 14 to 19 % DW, while FAME total lipid content determined by GC/MS was 7–11 % DW. The levels of TAG measured by GC/MS with TLC or HPLC–ELSD were similar, ranging from 3 to 5 % DW in *C. sorokiniana* CS-01 and UTEX 1230 which indicated the measurement of TAG was consistent between the different spectrometric methods. The failure of TAG detection under 1 % DW by GC/MS in *C. kessleri* UTEX 2229 may be contributed to the limited detection by the GC/MS and TLC method in *Chlorella* while the profile of lipid classes can be detected at 1–2 % DW of TAG by the HPLC–ELSD methods. Furthermore *C. sorokiniana* UTEX 1230 and CS-01 provided stable TAG contents in the large-scale 80 L aquarium culture but not *C. kessleri* UTEX 2229. Therefore *C. sorokiniana* UTEX 1230 and CS-01 might be better strains than *C. kessleri* UTEX 2229 for biofuel application.

### Comparison of the Time Courses of the TLC/GC and HPLC–ELSD Methods

TAG has been widely separated and quantified by the TLC and GC methods [3, 5, 44] and studied recently by using ESI–MS [45, 46] and LC/MS [47]. The conventional TLC/GC method was compared to the HPLC–ELSD method for TAG quantitation. Sample preparation using the TLC/GC method requires on average 4.8 h compared to 1.3 h using the HPLC–ELSD method. TAG amounts determined by HPLC–ELSD were comparable to those obtained by TLC/GC, provided that sufficient starting material was used so that the TAG measured by HPLC–ELSD is in the linear range of detection. After lipid extraction, HPLC–ELSD is a one-step procedure to detect and quantify the level of TAG and therefore efficient for high-throughput screening for lipid content in various and abundant algae under different conditions. Furthermore as the HPLC–ELSD method was established for separation and quantification of algal TAG which is chemically similar to most conventional fuels, it will take a great advantage for biofuel research and lipid analysis. For purposes of screening candidate algae and for determining optimal growth conditions for high oil yields, HPLC–ELSD provides a rapid and simple method for monitoring patterns of lipid accumulation in algae both of laboratory strains such as *Chlamydomonas* and industrially relevant strains such as *Chlorella*.

## Abbreviations

Ave.: Average
Conc.: Concentration
DAG: Diacylglycerol(s)
DGDG: Digalactosyldiacylglycerol
DW: DW
EI: Electron ionization
ELSD: Evaporative light scattering detector
ESI: Electrospray ionization
FAME: Fatty acid methyl ester(s)
HPLC: High performance liquid chromatography
GC: Gas chromatography
LC: Liquid chromatography
MGDG: Monogalactosyldiacylglycerol
MS: Mass spectrum
PtdCho: Phosphatidylcholine
RSD: Ratio of standard deviation
SD: Standard deviation
TAP: Tris acetate phosphate
TAG: Triacylglycerol(s)
TIC: Total ion count
TLC: Thin layer chromatography
